# Multi-site harmonization for magnetoencephalography spectral power data

**DOI:** 10.1162/IMAG.a.1099

**Published:** 2026-01-20

**Authors:** Allison C. Nugent, Anna M. Namyst, Frederick W. Carver, Paul M. Thompson, Jeffrey D. Stout

**Affiliations:** Magnetoencephalography Core Facility, National Institute of Mental Health, National Institutes of Health, Bethesda, MD, United States; Imaging Genetics Center, Mark & Mary Stevens Institute for Neuroimaging and Informatics, Keck School of Medicine, University of Southern California, Los Angeles, CA, United States

**Keywords:** magnetoencephalography, harmonization, ComBat, generalized additive models, resting-state, spectral power

## Abstract

A known issue with multi-site studies is the presence of site-specific effects that may confound effects of interest. These effects may be additive, multiplicative, or both. Numerous strategies have been developed and tested on microarray data from multiple batches, structural magnetic resonance imaging (MRI), diffusion tensor imaging (DTI), and functional MRI (fMRI). Multi-site magnetoencephalography (MEG) data represent a unique problem, however. The major MEG platforms differ substantially in sensor geometry, sensor layout, and noise-cancellation strategy, all of which may affect the distribution of the data. Another factor to consider in harmonization is retention of the relationship between the data and any covariates of interest. These relationships may be nonlinear, and individual sites may differ in the distribution of covariates. In this report, we test several previously developed methods for harmonization on a set of 16 open access datasets. We investigated ComBat, which uses empirical Bayes to improve model estimation; GAM-ComBat (Neuroharmonize), which extends ComBat to incorporate generalized additive modeling of the covariates of interest; CovBat (with the GAM extension), which performs a second round of ComBat harmonization to harmonize the covariance; and RELIEF, a matrix factorization technique. We found that overall, GAM-ComBat was the best choice for harmonizing the data while retaining the nonlinear dependence of the data on covariates of interest such as age. We demonstrate that harmonization of MEG data is possible and should be an integral part of any multi-site study.

## 1 Introduction

Recently, researchers in neuroimaging have begun to recognize that many published studies are inadequately powered and prone to irreproducibility (Poldrack et al., 2017). One potential solution to this is to collect very large single-site datasets, as has been done by the Human Connectome Project (Van Essen et al., 2012), although the collection of very large numbers of subjects at a single site may be logistically difficult without dedicated scanning facilities. Another alternative is to combine datasets collected at multiple sites into large mega- or meta-analyses. This has been done with several consortia, such as the Alzheimer’s Disease Neuroimaging Initiative (ADNI; Jack et al., 2010) and the Adolescent Brain Cognitive Development (ABCD) study (Casey et al., 2018). While ABCD and ADNI used streamlined data acquisition protocols, other initiatives, such as the ENIGMA consortium, have brought together disparate datasets collected as part of unrelated protocols at many sites to amass extremely large datasets, with a recent study incorporating data from over 70,000 participants (Garcia-Marin et al., 2024). While some ENIGMA studies use a meta-analytic framework, with all analyses carried out independently at each site, increasingly ENIGMA working groups are turning to mega-analysis as more statistically powerful (Zugman et al., 2022). This is enabled by the increasing willingness of groups to share raw data.

Large, multi-site studies have the advantage of great statistical power and are likely to produce more reliable and replicable results than small underpowered studies. Crucially, small sample sizes are prone to inflated effect sizes, potentially distorting power estimates (Cremers et al., 2017), and true effect sizes may be significantly lower than the often used value of d = 0.8 for a “large” effect size (Poldrack et al., 2017), increasing the required sample sizes for even 80% power into the hundreds. Power calculation in MEG is further complicated by differing sensitivity in different brain locations (Chaumon et al., 2021). Investigations into power and sample size in neuroscience uniformly indicate the need for larger sample sizes and multi-site investigations. Yet, these studies come with their own challenges. Even in well-controlled studies such as ADNI, where scan acquisition parameters were uniform, significant additive and multiplicative site effects have been demonstrated (Beer et al., 2020). Therefore, to avoid confounding the data with site effects, and to maximize statistical power to detect effects of interest, data harmonization must be performed. We can define data harmonization as the process by which effects of the data collection site are removed from the composite datasets, while retaining all effects of interest. Conceptually, additive site effects reflect differences in mean values across sites (potentially after correction for covariates), while multiplicative site effects reflect differences in variance of the measurements across sites.

A simple, first order correction to the issue of site effects is to simply model site as a random effect during statistical modeling. This can be problematic if effects of interest (such as age) differ significantly across sites. Given that this is frequently the case in multi-site data, this sort of correction is rarely sufficient. In addition, modeling site effects, particularly if site-specific variance is modeled, can add significantly to the complexity of the model and affect estimability. In addition, formal harmonization has been shown to out-perform random effects modeling in detection of effects of interest (Radua et al., 2020). Numerous techniques have been developed to perform harmonization as a separate step from modeling, perhaps the first of which was ComBat (Johnson et al., 2007). ComBat was originally developed to deal with batch effects in microarray data, and has subsequently been shown to be effective for harmonizing diffusion tensor imaging (DTI) data (Fortin et al., 2017), cortical thickness measurements (Fortin et al., 2018), and 3D diffusion tractometry (Chandio et al., 2024). It has also been shown to reduce site effects in the brain hemodynamic response function (Dole et al., 2023), surface and voxel-based morphology measures (Ma et al., 2023), structural connectivity (Chu et al., 2024), single-photon emission computed tomography (SPECT) (Wakasugi et al., 2024), and positron emission tomography (PET) (Zounek et al., 2023). There have been numerous extensions to ComBat, as well as alternative harmonization strategies. An important extension to the original algorithm was to develop a ComBat method for harmonizing longitudinal data (Beer et al., 2020). Particularly notable extensions are GAM-ComBat: the addition of generalized additive models to obtain data-driven relationships between outcome measures and covariates, as implemented in the neuroHarmonize package (Pomponio et al., 2020), and CovBat: designed to additionally correct differences in covariance across sites (Chen et al., 2022). GAM-ComBat has been used extensively in the literature to harmonize measures such as cortical thickness (Saponaro et al., 2022; Sun et al., 2022), DTI-derived white matter microstructure measures (Zhu et al., 2025), and MRI-derived brain structure volumes (Saponaro et al., 2022).

Other neuroimaging harmonization methods have been developed that operate on the raw images themselves; these include regression and normalization techniques such as RAVEL (Removal of Artificial Voxel Effect by Linear regression; Fortin et al., 2016) and WhiteStripe (Shinohara et al., 2014), and deep learning-based methods such as CALAMITI (Contrast Anatomy Learning and Analysis for MR Intensity Translation and Integration; Zuo et al., 2021) and MISPEL (Multi-scanner Image harmonization via Structure Preserving Embedding Learning; Torbati et al., 2023). (For further review of deep learning voxel-based methods, see Bayer et al., 2022.) Deep learning methods utilizing adversarial methods have also been explored for producing scanner-invariant images (Moyer et al., 2020), or to perform harmonization in the context of prediction tasks (Chattopadhyay et al., 2024; Dinsdale et al., 2021).

To our knowledge, there have been no demonstrations of data harmonization using MEG data, although there have been some attempts to harmonize electroencephalography (EEG) data. The simplest efforts at harmonizing EEG data collected at multiple sites with multiple systems involve resampling data to a common template and reference (Prado et al., 2023; Zanola et al., 2024), although these techniques do little to account for intrinsic site effects. One effort at combining multi-site quantitative EEG (qEEG) data (Li et al., 2022) used a data-driven approach to test multiple harmonization models, ultimately incorporating multivariate non-parametric functional dependence on biological variables, along with mean and variance effects for sex and batch. Parameters were estimated using a nonparametric multiple multivariate kernel regression. A more recent study using resting-state EEG data examined several methods, including ComBat, GAM-ComBat, an iterative nesting ComBat method utilizing Gaussian Mixture Models (OPNComBat-GMM), and HarmonizR, a variant of ComBat that compensates for missing data (Jaramillo-Jimenez et al., 2024). That study ultimately found that OPNComBat-GMM outperformed other methods at minimizing cross-site distances, while all methods successfully recovered linear relationships of spectral measures with age. No nonlinear covariate effects were examined, however.

Multi-site MEG has special challenges that may contribute to site effects. While it is common for multi-site MRI studies to integrate data from multiple scanning platforms, there are unique considerations with combining MEG data from multiple platforms. Systems may be equipped with magnetometers, axial gradiometers, planar gradiometers, or combinations of sensor types. These sensor types differ in their spatial sensitivity to current sources in the brain. Although sensor types can be transformed using mapping functions (as implemented in MNE Python), sensor layouts also vary substantially between systems. Thus, any combination of data from multiple platforms may best be performed after data have been projected into anatomical source space. Nevertheless, differences in statistical properties may remain. Different vendors also use different strategies for correcting for ambient noise, with MEGIN/Elekta systems using a software-based method (Taulu & Simola, 2006), while other manufacturers rely on reference sensors placed at some distance from the primary sensors. While these methods correct for most ambient magnetic noise, no correction scheme is perfect, and differences between sites in ambient noise magnitude and spectral content will contribute to site effects. Even within a site, there may be systematic differences between individual studies conducted by different research groups, potentially due to differences in subject preparation or positioning, or differences in ambient noise due to ancillary equipment present. Measuring supine versus seated can change which portions of the brain are closest to the MEG sensors, systematically impacting noise distribution across the brain. Even subtle effects such as the amount of padding or where it is placed could systematically alter the distribution of noise in a similar fashion. This is an important distinction between MEG and EEG, where positioning is less of a concern. Importantly, site differences may manifest differently across different parts of the frequency spectrum. We hypothesize that some parameters commonly measured by MEG would be less affected by site effects, such as peak alpha frequency. It is possible, however, that differences across sites in variance in the alpha portion of the spectrum could affect the fitting of these parameters. Likewise, we would expect latency values to also be less impacted by site effects, although the underlying temporal resolution of the time series could play a role. Furthermore, while metrics derived from within-subjects calculations have a natural correction for bias, some residual site effects may nonetheless remain. For example, while difference measures may have reduced additive site effects, multiplicative site effects may remain.

Crucially, as noted previously, any harmonization method must preserve effects of the covariates of interest. A particular motivation of the ENIGMA MEG working group is to determine the relationship between age and spectral power, and how the spatial distribution of spectral power changes across the lifespan. Two recent large studies to investigate lifespan effects using MEG have demonstrated profound nonlinear effects of age on spectral power (Doval et al., 2024; Rempe et al., 2023), emphasizing the need for harmonization methods that will accommodate nonlinear relationships. In particular, the largest of these studies found compelling evidence for cubic relationships in many regions (Doval et al., 2024). While the purpose of this report is not to demonstrate lifespan effects in spectral power, it is useful to review that in most studies, delta and theta power have been shown to decrease across the lifespan, while beta and gamma power typically increase (Doval et al., 2024; Gomez et al., 2013; Hardy et al., 2024; Hunt et al., 2019). Findings for alpha power have been more discordant, with studies showing that alpha power increases, decreases, or varies by brain region (Doval et al., 2024; Gomez et al., 2013; Hardy et al., 2024; Hunt et al., 2019; Rempe et al., 2023).

In this report, we describe the first attempt, to our knowledge, to apply statistical harmonization techniques to MEG data. As evidence mounts for a reproducibility crisis in neuroimaging (David et al., 2013; Nee, 2019; Poldrack et al., 2017), largely due to underpowered studies, very large-scale investigations are needed. While there are large single-site MEG studies, such as Cam-CAN (over 600 participants; Taylor et al., 2017), and several multi-site studies such as MEG-UK (over 400 participants), BioFIND (324 participants; Vaghari et al., 2022), and Dev-CoG (over 200 subjects; Stephen et al., 2021), none approach the numbers available in large multi-site MRI studies. In this manuscript, we will investigate four different algorithms for data harmonization in a combined dataset from 16 studies at 12 sites. These datasets were curated as part of an ongoing ENIGMA MEG Working Group project. Resting-state datasets will be analyzed to obtain measures of relative oscillatory power in five canonical frequency bands. We evaluate the raw data for the presence of study effects and evaluate the harmonization methods in terms of how well they remove these effects, while preserving dependence on biological covariates of interest. Notably, we expect that power will depend non-linearly on age of the participant, and we will determine whether these effects are preserved. The demonstration of data harmonization in MEG data has important implications for future multi-site studies.

## 2 Methods

### 2.1 Datasets

To test the harmonization techniques, we used a set of shared datasets. From the MEGUK project (MEG-UKI), we included two datasets collected at Aston, as well as data collected at Cambridge, Cardiff, Nottingham, and Oxford. We also included the MEG dataset from the Human Connectome Project (HCP; Larson-Prior et al., 2013), the Mother of Unification Studies (MOUS; Schoffelen et al., 2019), the Open MEG Archive (Omega; Niso et al., 2016), the National Institutes of Mental Health (NIMH) Research Volunteer dataset (Nugent et al., 2022), NatMEG-PD (Vinding et al., 2024), Cam-CAN (Taylor et al., 2017), the University of Pittsburg Aging Connectome project (Cohen et al., 2021), a combined dataset from Nebraska Boys Town (Rempe et al., 2023), and data from an NIH study on adolescent anxiety (Liuzzi et al., 2021). From each of these datasets, individual MEG recordings and MRI scans were processed through the ENIGMA MEG pipeline (Stout et al., 2025; Nugent et al., Under Review). For the purposes of this analysis, only healthy control datasets were included. This analysis represents a subset of our larger ENIGMA project, which was reviewed by the NIH Combined IRB and was given a non-human subjects research determination.

### 2.2 Initial processing

All processing was performed using the ENIGMA MEG pipeline (Stout et al., 2025) and (Nugent et al., Under Review). First, the recon-all procedure in FreeSurfer (Dale et al., 1999; Fischl et al., 1999) is used to process a T1-weighted structural MRI and obtain cortical surfaces, as well as the 448 parcel sub-parcellation of the Desikan–Killiany atlas (Desikan et al., 2006; Khan et al., 2018). The MEG recordings are preprocessed to remove bad channels and vendor-specific noise cancellation is performed. Artifact detection and removal are performed in an automated fashion using a re-trained version of MEGnet, a convolutional neural network model (Stout, 2025; Treacher et al., 2021). Next, resampling, filtering, and epoching are performed, and the data are projected into source space using a linearly constrained minimum variance (LCMV) beamformer. From each parcel, a summation of the power spectral density is calculated on the first 15 principal components of all vertices in the parcel. Relative power is calculated in five canonical bands: delta (1–3 Hz), theta (3–6 Hz), alpha (8–12 Hz), beta (13–35 Hz), and gamma (35–45 Hz). For the purposes of demonstration of the harmonization techniques, groups of parcels in the sub-parcellation are averaged to recover the original 68 DK atlas parcels. For each site, and for each canonical band, a matrix is assembled with S rows (where S is the number of subjects) and columns for each covariate and parcel (feature). Included covariates were sex, task (eyes open/eyes closed), and age. For ComBat, age squared and age cubed were additionally included. Note that study was used as the grouping variable rather than site. This was done for two reasons. First, it would allow harmonization of differences due to experimental methods that may have differed between studies at a given site. Second, it further allows the demonstration of the ability of the harmonization methods across studies with differing demographic distributions.

### 2.3 Harmonization methods

Four different harmonization methods were tested. First, we tested a Python implementation of the original ComBat algorithm (Fortin et al., 2017, 2018). ComBat methods start with a general assumption about the structure of the data and site effects, representing the measured outcome data for each scan j at site i in each voxel (or in this case parcel) v, as

$$y_{ijv}=\;\alpha_{v}+\;{\text{X}}_{ij}\beta_{v}+\;\gamma_{iv}+\;\delta_{iv}\varepsilon_{ijv},$$

where $\alpha_{v}$ is the overall outcome measure in parcel n, ${\text{X}}_{ij}$ is the design matrix containing the covariates of interest, and $\beta_{v}$ is the set of unique regression values for each parcel (note we follow the notation in Fortin et al. (2018)). Importantly, this assumes a linear effect of the covariates of interest. The additive site effects are represented by $\gamma_{iv}$ , while the multiplicative site effects are represented by the term $\delta_{iv}$ , which is multiplied by the standard error term $\varepsilon_{ijv}$ , with mean zero and variance $\sigma_{v}^{2}$. ComBat estimates an empirical distribution for the additive and multiplicative site effect terms, and assumes that site effects from all parcels come from a common distribution. The additive site effects are modeled as a normal distribution, while the multiplicative effects are modeled using an inverse Gamma function. The empirical Bayes process is then used to improve the estimates of the site effects, using a shrinkage method to draw the site parameters toward the prior estimates derived from the pooled sample. Finally, the harmonized data are represented as follows:

$$y_{ijv}^{ComBat}=\;\frac{y_{ijv}-\;{\hat{\alpha }}_{v}-\;{\text{X}}_{ij}{\hat{\beta }}_{v}-\;\gamma_{iv}^{*}}{\delta_{iv}^{*}}+\;{\hat{\alpha }}_{v\;}\text{​}+\text{​}\;{\text{X}}_{ij}{\hat{\beta }}_{v}.$$

The overall parcel effect, the effects of covariates, and the additive site effects are subtracted from the original data and divided by the multiplicative site effects. Then the overall effect and covariate effect are added back to produce the final harmonized value.

The second method we examine is the GAM-ComBat extension, available as the neuroHarmonize package (Pomponio et al., 2020). In this framework, the term $\alpha_{v}+\;{\text{X}}_{ij}\beta_{v}$ is replaced by a generalized additive model (GAM), which is a function of the covariates of interest but informed by the data. The underlying relationship of the covariates to the data is represented using a basis expansion, with smoothing applied to avoid overfitting. Following estimation of the GAM model, harmonization with empirical Bayes optimization of parameters proceeds as in the original ComBat model.

The next method we examine is CovBat, which attempts to additionally mitigate site effects in the covariance of multi-site data (Chen et al., 2022). The first step of the CovBat procedure is to perform ComBat. For this first stage, we chose to perform GAM-ComBat rather than the original version of ComBat. Following this first step, the residuals are defined as follows:

$$e_{ijv}^{ComBat}=\;\frac{y_{ijv}-\;{\hat{\alpha }}_{v}-\;{\text{X}}_{ij}{\hat{\beta }}_{v}-\;\gamma_{iv}^{*}}{\delta_{iv}^{*}}.$$

In step 2, a PCA is performed on the residuals, resulting in the following expression:

$$e_{ijv}^{ComBat}=\;\sum_{k=1}^{q}\;\xi_{ijk}{\hat{\phi }}_{k}.$$

where $\xi_{ijk}$ are the principal component scores, ${\hat{\phi }}_{k}$ are the eigenvectors of the sample covariance matrix, and q represents the rank. Next, ordinary ComBat is performed on the principal component scores $\xi_{ijk}$ , assuming further additive and multiplicative site effects as follows:

$$\xi_{ijk}\text{​}=\mu_{ik}+\;\rho_{ik}\varepsilon_{ijk}.$$

Through the empirical Bayes procedures, the $\mu_{ik}$ and $\rho_{ik}$ parameters are estimated, and

$$\xi_{ijk}^{CovBat}=(\xi_{ijk}-\;{\hat{\mu }}_{ik})/{\hat{\rho }}_{ik}.$$

The adjusted scores are then projected back into residual space. Importantly, the full rank of PC scores is not necessarily corrected; rather than harmonizing all PC scores only the first K can potentially be adjusted, as follows:

$$e_{ijv}^{CovBat}=\;\sum_{k=1}^{K}\;\xi_{ijk}^{CovBat}{\hat{\phi }}_{k}+\;\sum_{l=K+1}^{q}\;\xi_{ijk}{\hat{\phi }}_{l}.$$

Finally, the intercepts and covariate effects are added back to give the final harmonized values.

$$y_{ijv}^{CovBat}=\;e_{ijv}^{CovBat}+\;{\hat{\alpha }}_{v\;}\text{​}+\;{\text{X}}_{ij}{\hat{\beta }}_{v}.$$

Note that the choice of the tuning parameter K, which represents the amount of explained variance that is harmonized, is not straightforward, and the performance under different values is affected by sample size. After extensive simulations of differing mean and covariance effects, the authors of the original manuscript caution that residual site effects may be larger in CovBat harmonized data in some cases, especially where the number of samples per site is small and the number of features is large.

The final harmonization method tested is RELIEF (Zhang et al., 2023), which is conceptually distinct from the ComBat methods. The original data are conceptualized as a matrix sum, as follows:

$$Y=A+\beta X^{'}+[\Gamma_{1},\ldots ,\Gamma_{M}]+R+[I_{1}\ldots ,I_{M}]+[\delta_{1}E_{1},\ldots ,\delta_{M}E_{M}].$$

The matrix A contains the intercepts, $\beta X^{'}$ are the regression coefficients and known covariates, the Γ matrices are additive site effects, the R matrix contains shared variation across all scanners not explained by covariate effects and includes nonlinear and unknown covariate effects. The I matrices contain latent site effects (which may be nonlinear), and finally the δE matrices are noise matrices, with δ representing the variance heterogeneity across sites. First, $\hat{\text{A}}$, $\hat{\beta }$, and $\left[{\hat{\Gamma }}_{1};\ldots ;{\hat{\Gamma }}_{\text{M}}\right]$ are determined through a two-stage regression. Next, the residual matrix is scaled to obtain homogeneous variances across sites, and ${\hat{\text{R}}}^{*}$ and ${\hat{\text{I}}}^{*}$ are estimated using an iterative algorithm. Then, the final harmonized data are expressed as follows:

$${\text{Y}}^{\text{RELIEF}}=\hat{\text{A}}+\;\hat{\beta }{\text{X}}^{\prime }+\left[{\hat{\delta }}_{1}{\hat{\text{R}}}_{1}^{*};\ldots ;{\hat{\delta }}_{M}{\hat{\text{R}}}_{\text{M}}^{*}\right]+\hat{\delta }\;\hat{\text{E}}.$$

Of note, because the latent effect matrix R is preserved, the authors recommend against including any covariates explicitly in the model.

### 2.4 Evaluation of harmonization methods

The harmonization methods were evaluated in two domains: their ability to remove site effects and their ability to retain effects of interest. First, a linear regression was used to remove effects of sex, condition, age, age squared, and age cubed from the harmonized data. ANOVA was used to assess the presence of residual additive study effects, and Levene’s test of variances was used to assess the presence of residual multiplicative site effects. We do, however, acknowledge that there are limitations to this null hypothesis significance testing (NHST) framework. We could fail to reject the null hypothesis for a site effect simply if the sample sizes were too small (although we note that our sample size is quite large). In addition, where no significant effect of site is found using the NHST framework, it is difficult to judge performance between harmonization methods on the basis of the F values alone. We nonetheless report these values, as they provide a measure of the ratio of site-related variance to overall variance present in the harmonized data. Given the limitations of the NHST framework, and to provide a better measure of the amount of additive and multiplicative effects remaining in the data, we have performed an additional analysis which should further increase confidence in the efficacy of the harmonization. We performed two regressions of the data, one using all the relevant covariates (age, age squared, age cubed, sex, condition, and study) and another omitting the study effect. We then calculate the partial R^2^ value for the study effect in each parcel as a measure of efficacy of removal of additive study effects. To assess the removal of multiplicative site effects, we calculate the mean absolute log variance ratio (mean |logVR|) for each parcel by calculating the log of the ratio of the variance of the residuals in each site and the pooled variance of all residuals, averaged over all sites. We plot the distributions of the partial R^2^ values and mean|logVR| across sites and report their medians. Importantly, this analysis does not suffer from the same limitations as the NHST framework, and can provide meaningful comparisons between harmonization methods.

Next, we assess whether covariate effects are preserved. First, we compare relative alpha power in the eyes-open versus eyes-closed condition to verify the expected increased power in the eyes-closed condition. We performed this analysis first in a subgroup of six studies in which participants had both eyes-open and eyes-closed resting-state recordings; paired *t*-tests were used to assess differences in left and right lateral occipital cortex and left and right pericalcarine cortex. This analysis provides an estimate of how well harmonization incorporating multiple effects of interest preserves the effect of a single covariate within each study. Next, to assess whether harmonization enhances (or attenuates) covariate affects across studies, we perform an independent samples *t*-test in the same regions in our full dataset where each participant has only one (eyes open or eyes closed) recording. Finally, to assess the preservation of nonlinear covariate relationships, we fit quadratic and cubic curves to relative power versus age, giving R^2^ values for each parcel fit. We visualize the results for delta power, because it has been previously shown to have very large nonlinear effects of age (Doval et al., 2024; Rempe et al., 2023), specifically in a parcel previously identified to have a maximum age effect (Rempe et al., 2023). We also compare harmonization methods using paired cross-validation ∆R^2^. We used a 10-fold cross-validation repeated 50 times. The ∆R^2^ differences between methods for each fold were compared with paired t-tests, FDR corrected (Benjamini–Hochberg) across all parcels. Because this is done in a pairwise fashion, only two methods can be compared at a time.

### 2.5 Illustration of harmonization efficacy on brain maps

First, we plot the ANOVA F tests and Levene’s test of equality of means across the brain, for the raw data and for the harmonized data using one of the best performing methods and one of the worst performing methods, separately for all spectral power bands. This is intended to illustrate both the spatial distribution of site effects and the spatial distribution of the corrections. It is also useful to plot the spectral power on the brain pre- and post-harmonization. However, this is difficult due to the dependance of spectral power (and the spatial distribution of spectral power) on age, sex, and condition (eyes open/eyes closed), and the fact that our studies differ in their distributions on those variables. Therefore, we chose to plot raw and harmonized data for a single age group (20–30 years of age) for 7 studies that are well represented in that age group (CamCAN, Cardiff, HCP, MOUS, NIHHV, Oxford, and REMPE). We do this for the raw data and our best performing harmonization method. We then compare the concordance of these brain maps across sites using several metrics. We calculate pairwise Pearson R correlation coefficient between sites and report the median; we calculate concordance correlation coefficient (CCC) and report the median; we calculate and report intraclass correlation across the sites (ICC(3,k); two-way mixed, absolute agreement, average of raters); and we report median coefficient of variation across parcels (CV). Because these values do not represent all sites and all ages, we do not use them to evaluate all harmonization methods. We do expect some between-site differences will remain post-harmonization, potentially due in part to other variables (i.e., sex/condition).

## 3 Results

### 3.1 Subjects

**Fig. 1.**
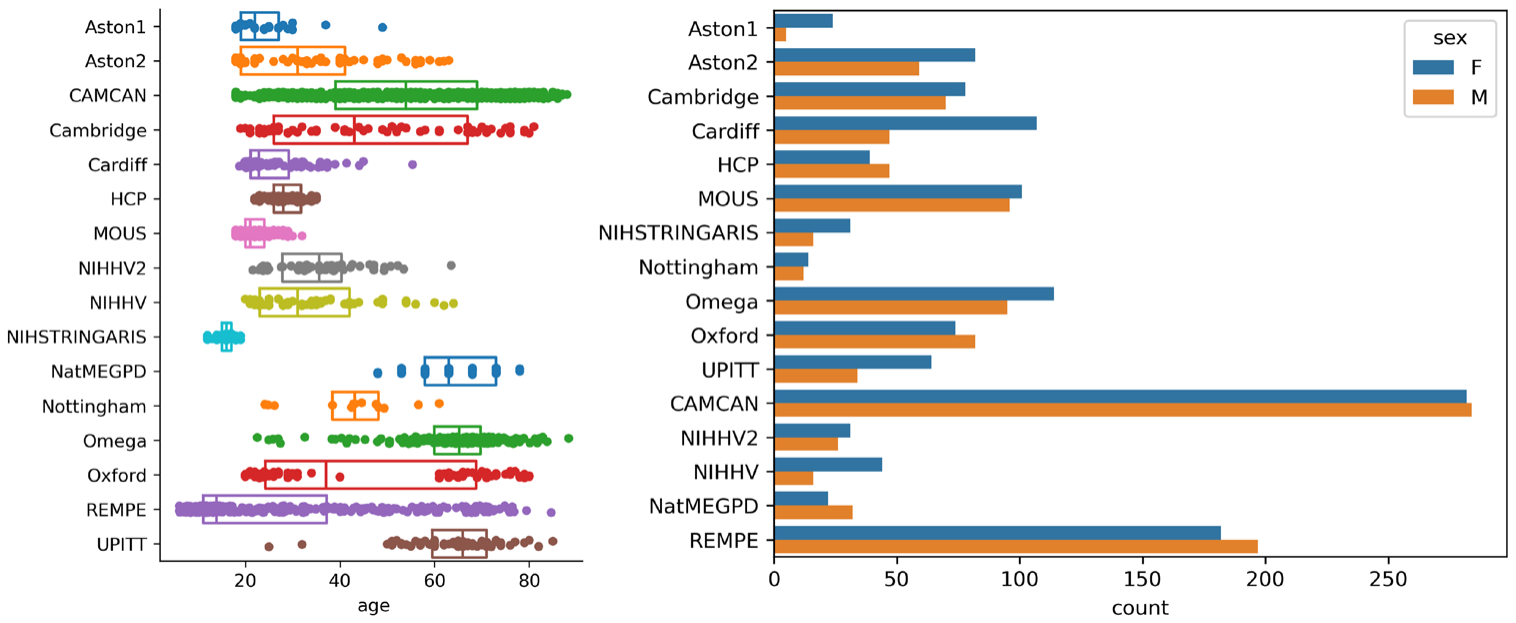
Demographic makeup of the 16 included studies by age and sex.

A total of 2,407 datasets were included in the harmonization procedure from 2,075 healthy participants, including 1,456 eyes-closed recordings and 951 eyes-open recordings. A subset of 332 participants (from 6 studies) had both eyes-open and eyes-closed recordings. For the purposes of assessment of removal of study effects, and examination of age effects, only a single recording was included from each subject. Because we had fewer eyes-open datasets overall, we retained the eyes-closed datasets for subjects with both, resulting in 1,124 eyes-closed recordings and 951 eyes-open recordings. Demographic distributions (including age and sex) are shown in Figure 1 for each included study.

### 3.2 Assessment of removal of study effects

**Fig. 2.**
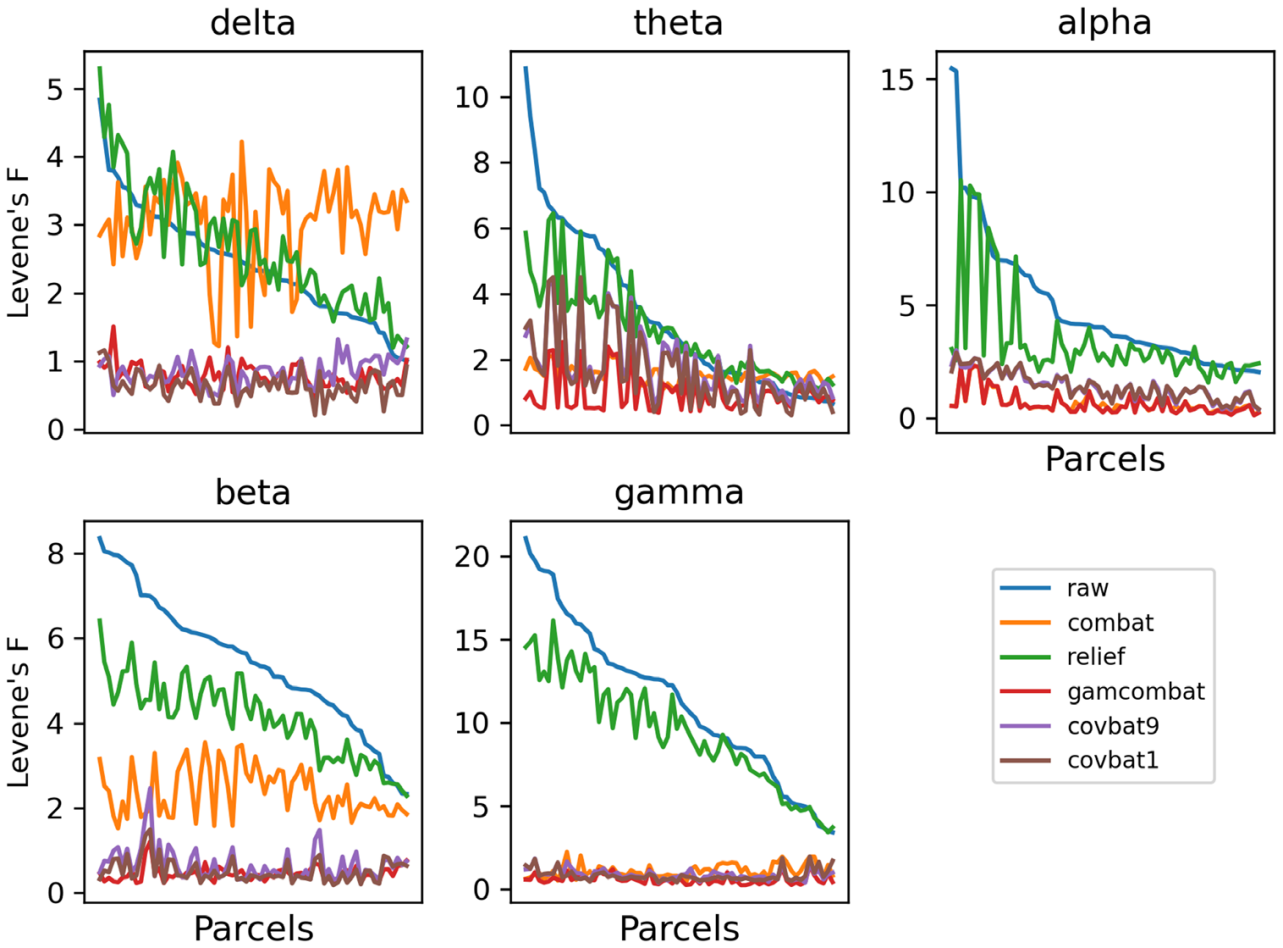
Results of Levene’s *F*-test for equality of variances across studies for residualized data, plotted for each parcel, for each frequency band (parcels sorted by site effect in the unharmonized sample). Differences in variance across sites were evident in the raw, unharmonized data for every frequency band. RELIEF produced poor control over study differences for all frequency bands, while ComBat performed poorly for delta and beta power.

**Fig. 3.**
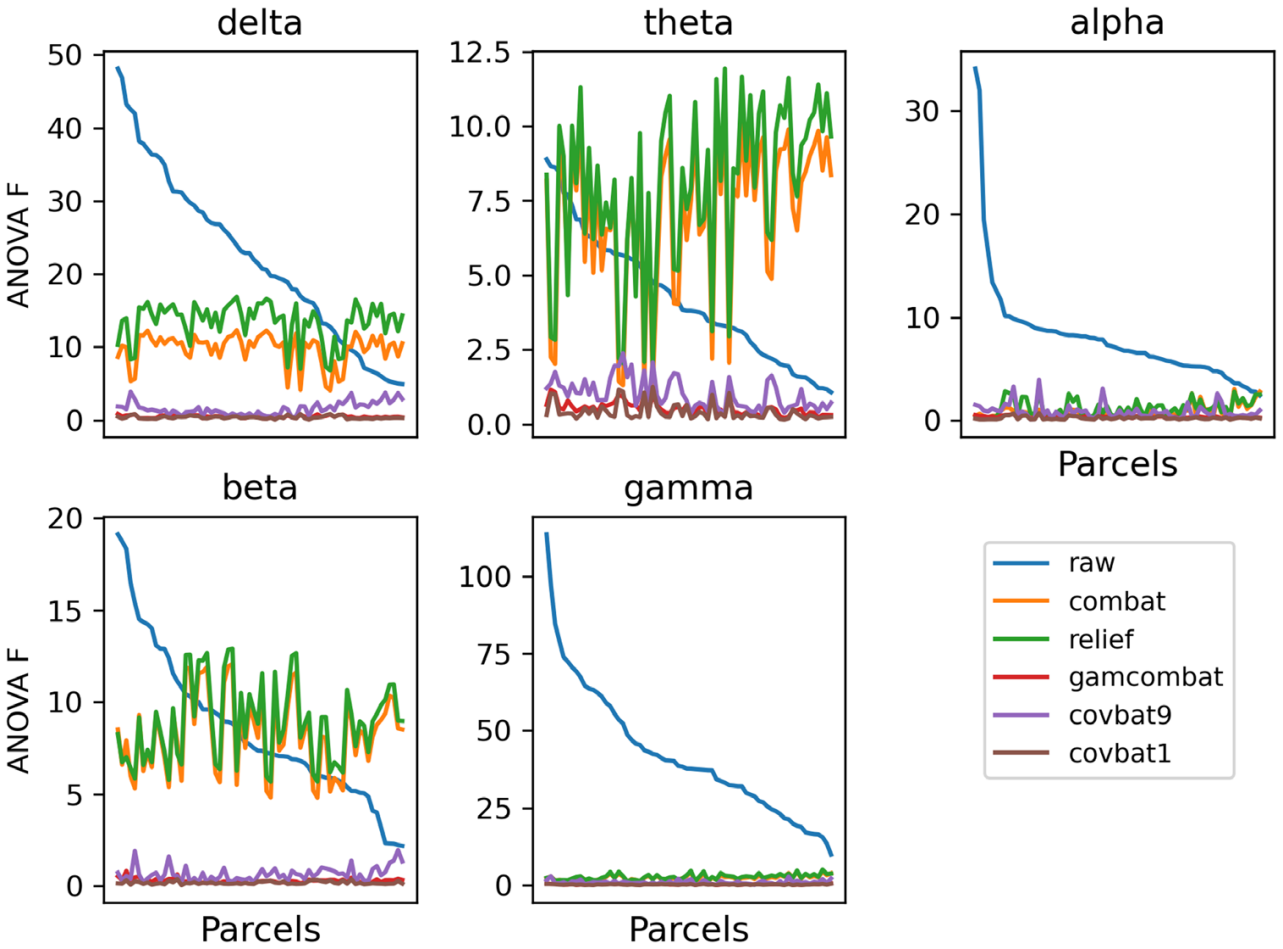
Results of ANOVA across studies for residualized data, plotted for each parcel, for each frequency band (parcels sorted by site effect in the unharmonized sample). As with variance, differences in mean values across site were evident in the raw data in all frequency bands. RELIEF and ComBat performed inconsistently, showing poor performance in delta, theta, and beta power.

**Fig. 4.**
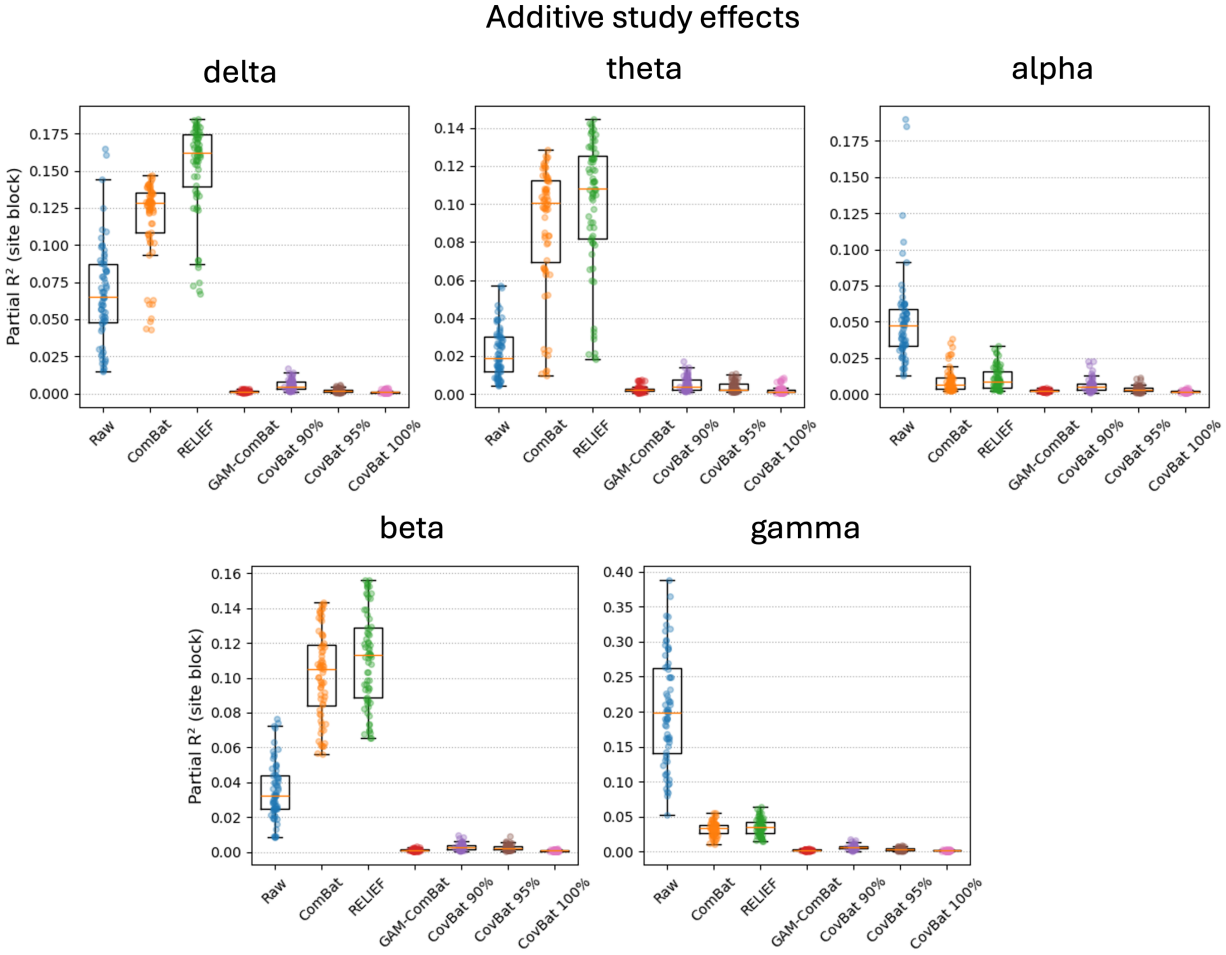
Illustration of additive study effects in the data, parametrized as the partial R^2^ value uniquely attributable to site. In delta, theta, and beta bands, ComBat and RELIEF amplified the partial R^2^. Values were similar for GAM-ComBat and all CovBat methods.

**Fig. 5.**
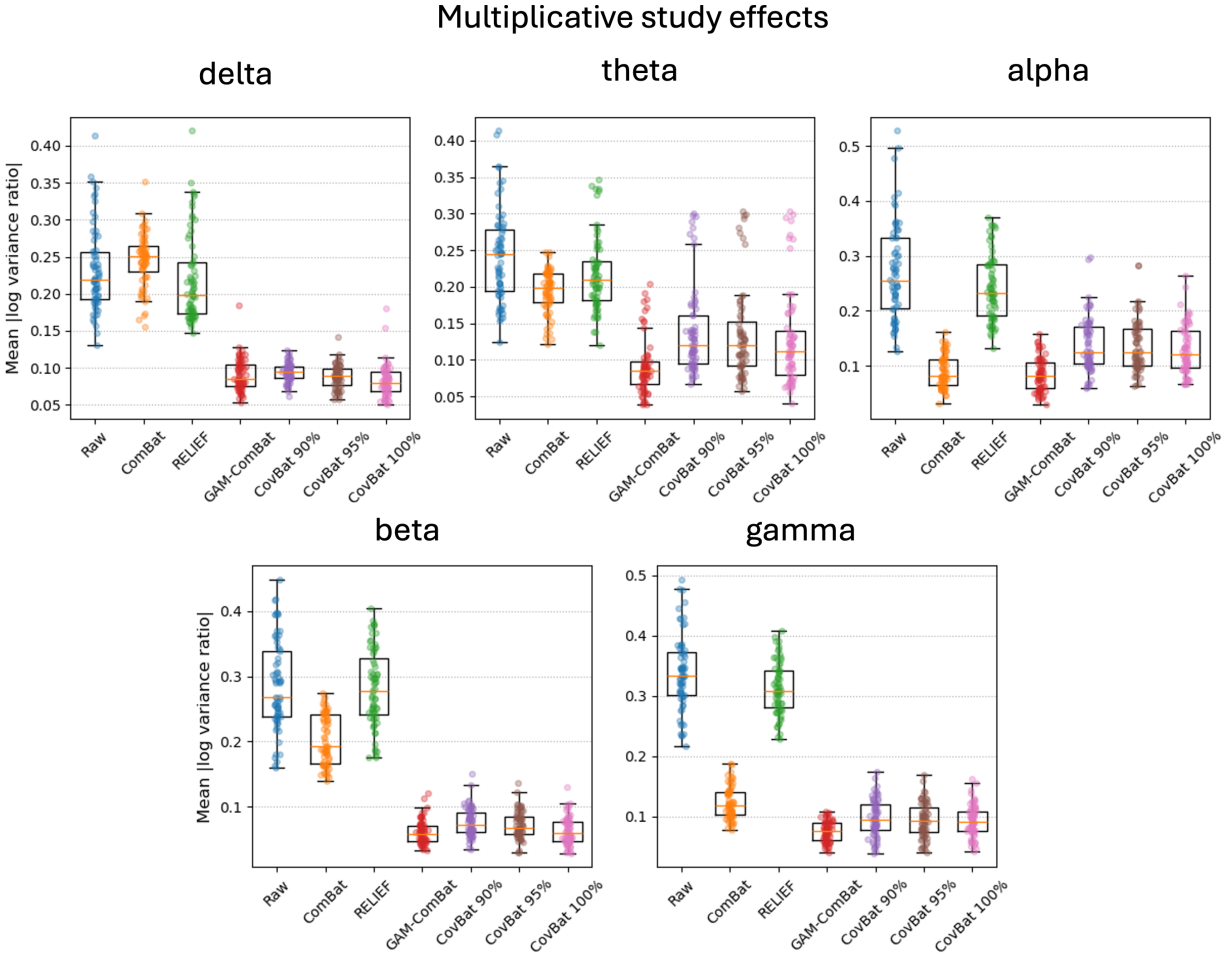
Illustration of multiplicative study effects in the data, given as the absolute log variance ratio (mean |logVR|). With the exception of delta power, GAM-ComBat consistently resulted in less residual multiplicative effects post-harmonization compared with the CovBat methods.

Raw and harmonized datasets were residualized using linear regression for sex, task, age, age squared, and age cubed. In Supplementary Figure S1, we show the residualized beta power in an exemplar region plotted categorically by study, to show how residual site effects present in the raw data are altered by the different harmonization methods. We then perform Levene’s test for equality of variances and ANOVA for equality of means with study as the group variable for each parcel. Mean test statistics over all parcels are given in Supplementary Table S1. In addition, test statistics are plotted for Levene’s test (Fig. 2) and ANOVA (Fig. 3) for all frequency bands for all correction methods (CovBat 95% is omitted for clarity) for all parcels. We note that here we sort the parcels according to the F test in the raw unharmonized sample, to descriptively illustrate the response of the methods to varying study effects across parcels. While F values do provide a metric of the remaining study-related variance compared with the overall unexplained variance, it is difficult to interpret small differences in F values across methods. Nevertheless, GAM-ComBat qualitatively performed better at reducing the differences in variances across study, and while CovBat 100% and GAM-ComBat qualitatively performed similarly at controlling mean differences, CovBat 100% performed slightly better. In Table 1, we give the R^2^ values for cubic fits, illustrating the improvement in goodness of fit for harmonized data. To provide a more quantitative estimate of the amount of residual additive and multiplicative study effects after harmonization, we present additional metrics. In Figure 4, we show the remaining additive site effects in the model, given by the partial R^2^ value attributable to the site effects for each harmonization method. Note that for some measures, the additive site effects appeared amplified in the post-harmonized data for the ComBat and RELIEF methods. Although median values were lowest for CovBat 100% (see Supplementary Table S2), GAM-ComBat and the three CovBat versions tested performed similarly. In Figure 5, we show the multiplicative effects, given as the log of the ratio of the study variance over the total pooled variance, averaged across sites. GAM-ComBat generally performed best at controlling multiplicative site effects.

**Table 1.** R2 goodness of fit values for cubic fits for each frequency band and for each harmonization method.

	R2 values for cubic age fit				
	delta	theta	alpha	beta	gamma
raw	0.507	0.354	0.035	0.374	0.099
combat	0.11	0.084	0.011	0.094	0.037
relief	0.142	0.092	0.009	0.01	0.033
gamcombat	0.527	0.347	0.035	0.379	0.144
covbat 90%	0.515	0.334	0.035	0.356	0.13
covbat 95%	0.518	0.334	0.035	0.356	0.13
covbat 100%	0.518	0.335	0.034	0.357	0.129

The highest values are highlighted in bold.

### 3.3 Assessment of retention of effects of interest

**Fig. 6.**
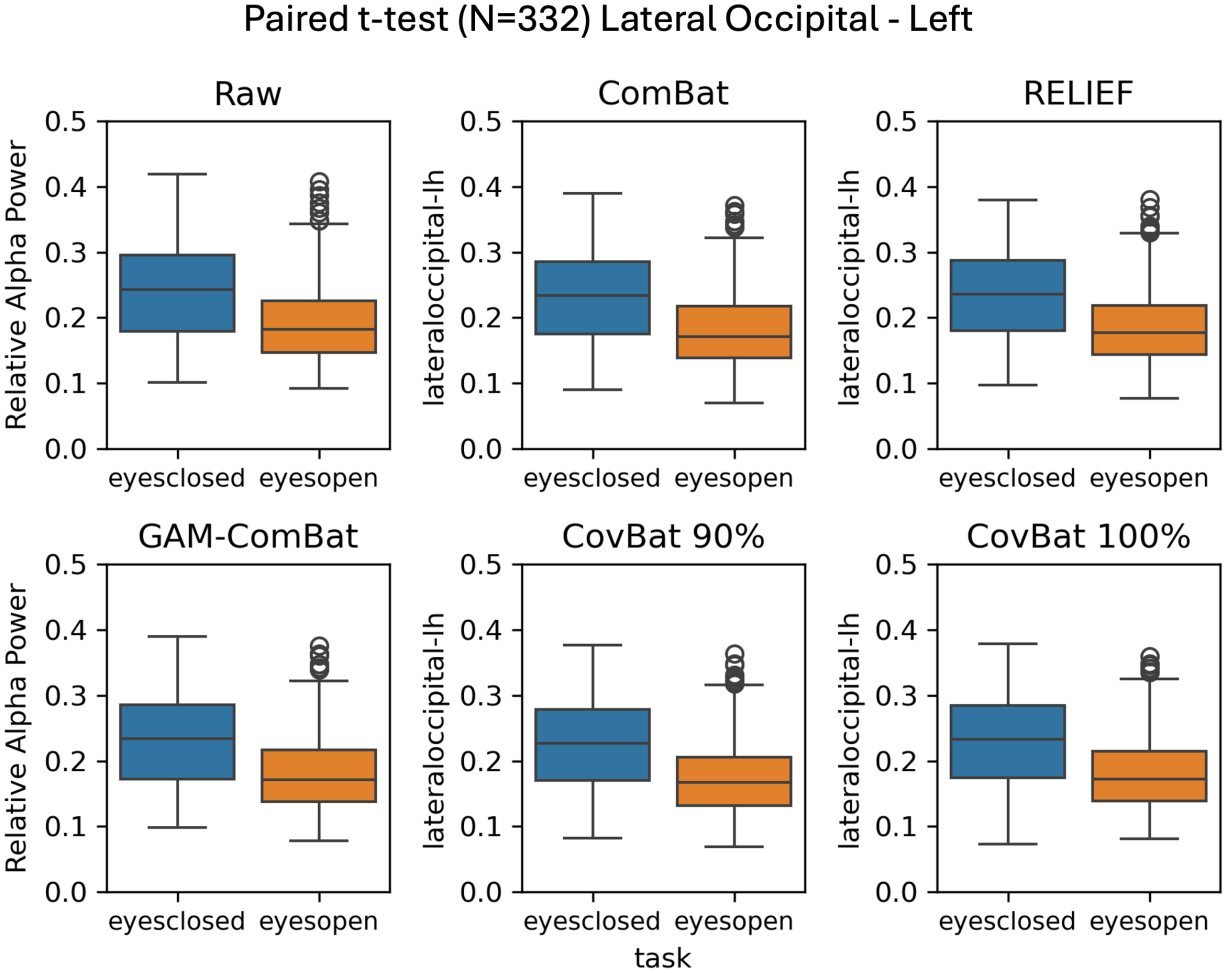
Illustration of preservation of task effects in participants (N = 332) who had both eyes-open and eyes-closed recordings (participants drawn from 6 studies). All harmonization methods retain the task effect post-harmonization in this within-subjects design.

**Fig. 7.**
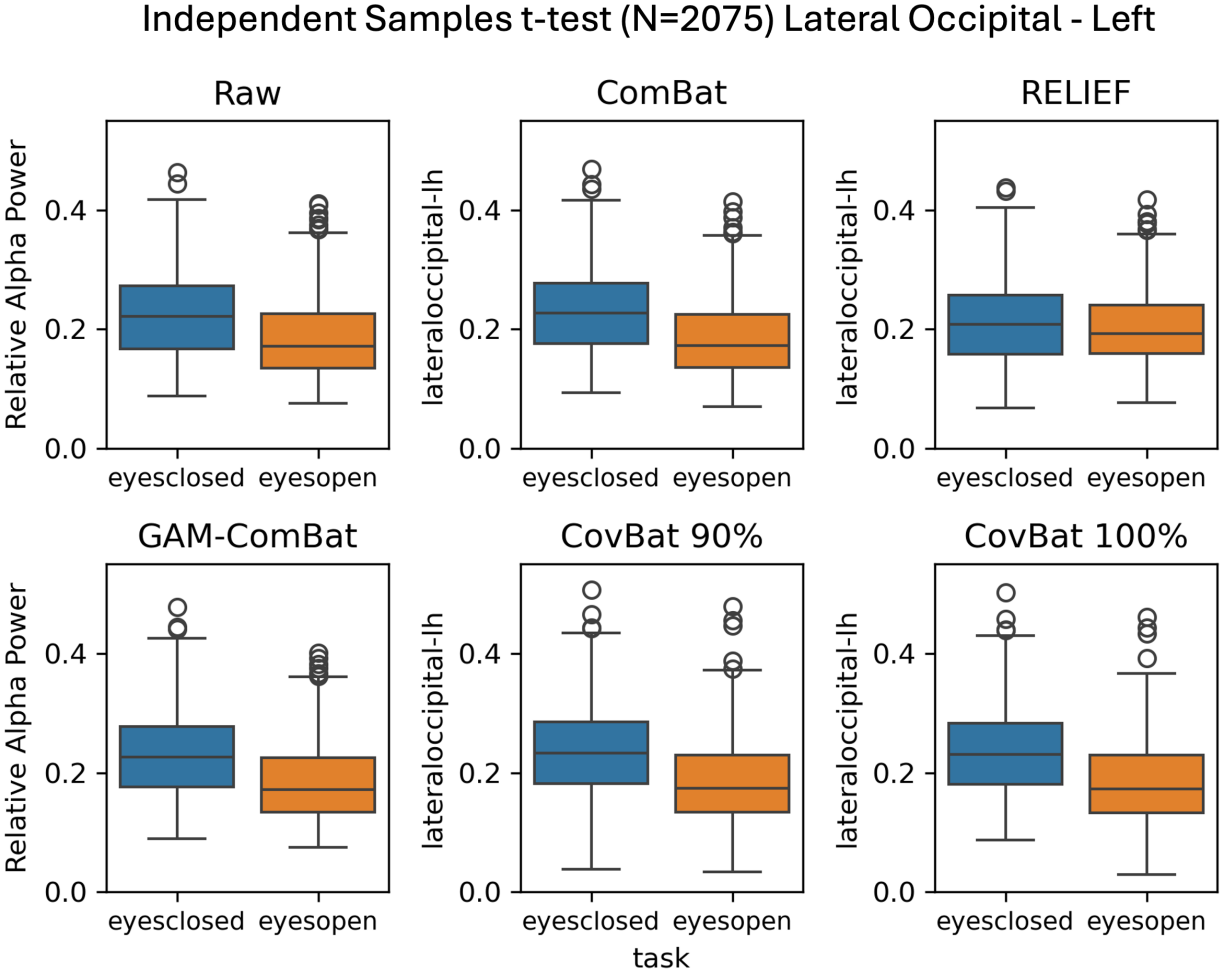
Illustration of preservation task effects in all participants (N = 2,075) where only one recording is included per subject. All harmonization methods, except for RELIEF, maintain task effects post-harmonization in this between-subjects design, although CovBat methods appear to enhance outliers.

We tested two effects of interest, age and task (eyes-open or eyes-closed resting). First, we examine the simple categorical effect of task in a subset of the data including only those participants (N = 332 from 6 studies) who had both eyes-open and eyes-closed datasets using paired *t*-tests, with results given in Table 2. In this within-subjects design, RELIEF produced the highest *t*-values in three out of four regions, although all methods tested preserved the effect. Figure 6 shows boxplots of eyes-open versus eyes-closed relative alpha power in the left lateral occipital cortex. Also in Table 2, we give the results of the independent samples *t*-test, where only one scan per participant was included (N = 2,075). In contrast to the paired *t*-test results, RELIEF performed poorly, indicating that while within-study covariate effects are preserved by this method, this across-study covariate effects are distorted by the harmonization. CovBat 95% performed the best across all regions, although all tested harmonization methods (except RELIEF) actually improved the detection of the change in alpha power in the eyes-open versus eyes-closed comparison. Comparing GAM-ComBat and CovBat 100%, our two best methods at controlling mean and variance study effects, GAM-ComBat uniformly produced larger t-values in both samples. In Figure 7, we show the boxplots of eyes-open versus eyes-closed relative alpha power in the left lateral occipital cortex for the between-subjects comparison.

**Table 2.** Table of *t*-values for the comparison of relative alpha power in the eyes-open versus eyes-closed resting state for four regions of interest.

	Relative alpha power, Paired t-tests, N = 332, eyes- open vs. eyes-closed			
	pericalcarine-lh	pericalcarine-rh	lateraloccipital-lh	lateraloccipital-rh
raw	*-14.01*	*-13.53*	*-14.11*	*-14.12*
ComBat	-13.50	-13.03	**-14.24**	-13.98
RELIEF	**-13.93**	**-13.49**	-14.00	**-14.00**
GAM-ComBat	-13.51	-13.06	-14.19	-13.98
CovBat 90%	-12.91	-12.48	-14.16	-13.76
CovBat 95%	-12.87	-12.45	-14.13	-13.72
CovBat 100%	-12.83	-12.37	-13.88	-13.50

	Relative alpha power, Independent samples t-tests, N = 2,075 eyes-open vs. eyes-closed			
	pericalcarine-lh	pericalcarine-rh	lateraloccipital-lh	lateraloccipital-rh
raw	*-13.79*	*-13.71*	*-13.22*	*-12.36*
ComBat	-15.82	-15.13	-16.41	-15.63
RELIEF	-2.92	-2.79	-3.06	-2.90
GAM-ComBat	**-16.24**	**-15.81**	-16.55	-16.74
CovBat 90%	-13.14	-13.11	-17.17	-16.62
CovBat 95%	-14.60	-14.44	**-18.51**	**-17.69**
CovBat 100%	-14.77	-14.64	-17.02	-16.12

The top half gives *t*-values for the subset of participants (N = 332 from 6 studies) who have both eyes-open and eyes-closed recordings; paired *t*-tests were used. The lower half gives *t*-values for the full set of participants with only one recording included per subject; independent samples t-tests were used. The *t*-values for the raw data are highlighted in italic and the highest *t*-value for each ROI across harmonization methods is highlighted in bold.

**Fig. 8.**
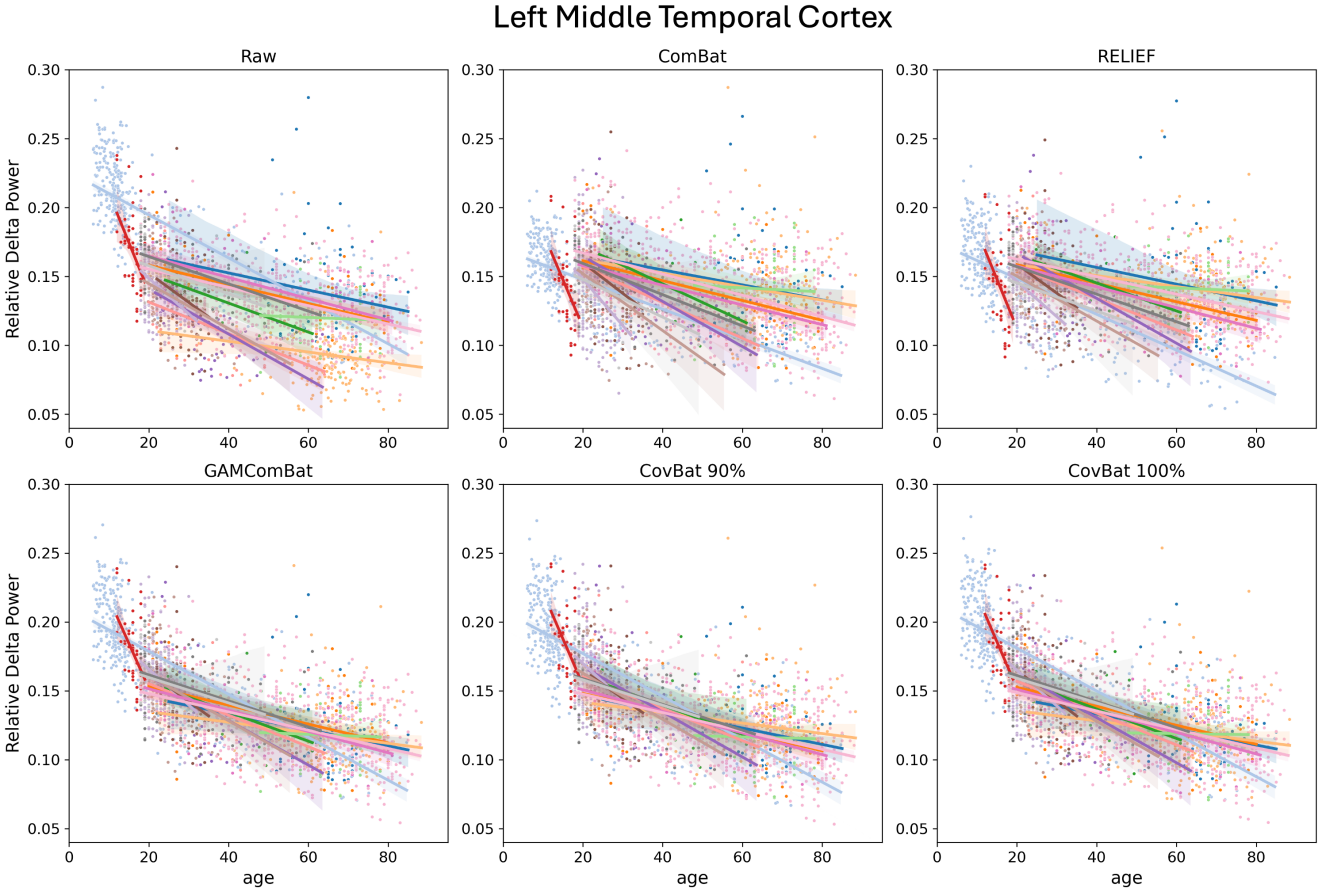
Scatter plots of raw and harmonized relative delta power in the left middle temporal cortex. Markers are color coded by study, and simple linear trend lines are shown to show the effects of harmonization.

**Fig. 9.**
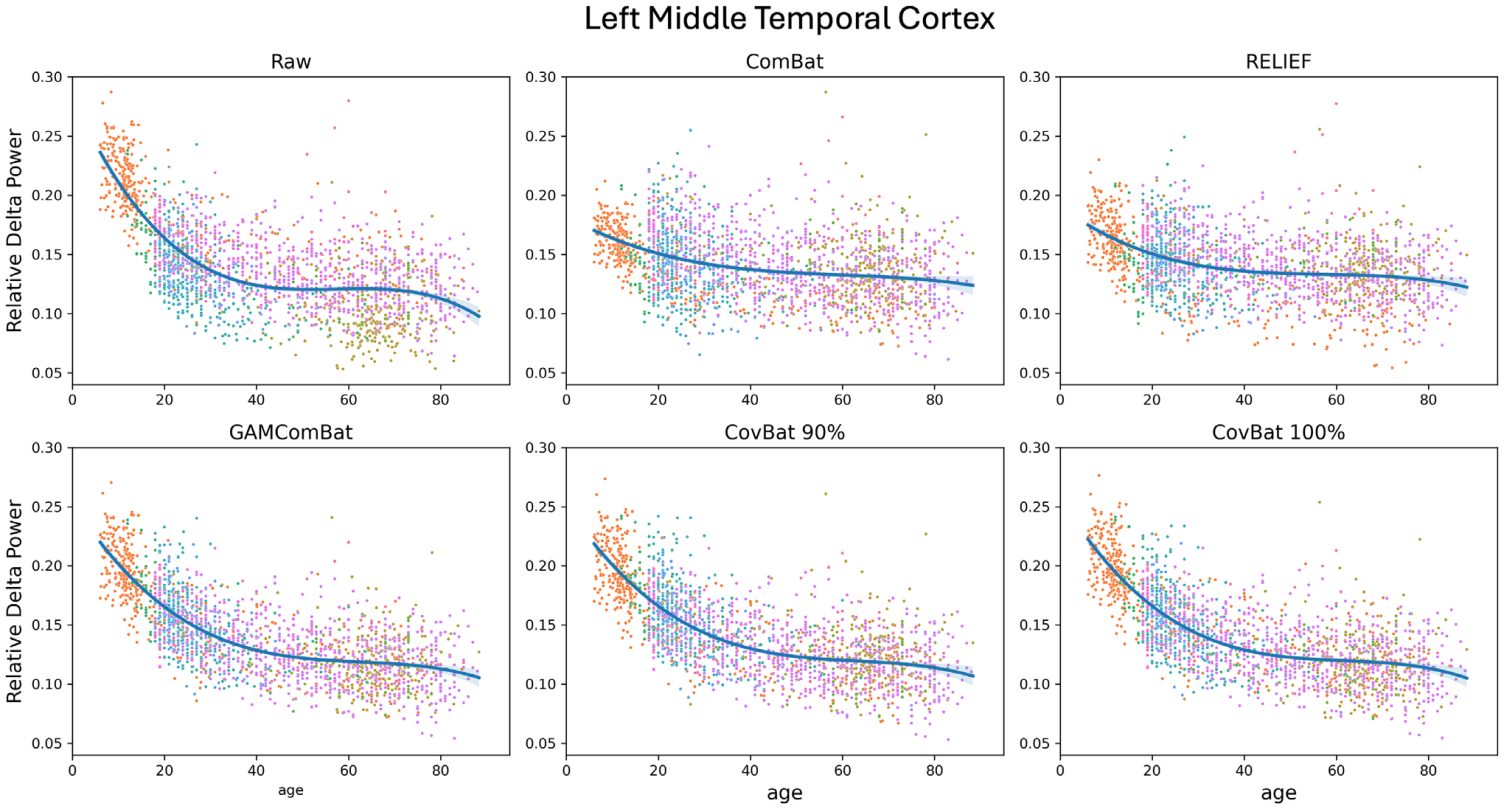
The same data as sown in Figure 7, but with an overall cubic fit. Markers in the scatterplot are color coded by study.

Finally, we examine the retention of age effects, which are known to be non-linear. Although we performed linear, quadratic, and cubic fits, given the evidence from the literature that cubic fits are likely superior for many regions, we present only those R^2^ values in Table 3 (although results are similar across all fits). In Figure 8, we show relative delta power plotted versus age in the left middle temporal cortex (a frequency band/cortical area previously shown to have large non-linear age effects; Rempe et al., 2023). For each study, a linear trendline is shown for convenience and to aid in visualization of the differences between the studies (because of the restricted age ranges of some studies, a non-linear fit was not possible for every study). In Figure 9, we show the same data, but with an overall cubic fit line for all studies combined. It is evident from these figures that ComBat and RELIEF perform quite poorly at retaining non-linear age effects. GAM-ComBat consistently produced the highest *R*^2^ values, likely reflective of the superior harmonization of variance across groups using this method. We chose to statistically compare GAM-ComBat against CovBat-100% using paired cross-validation ∆R^2^. For delta, theta, beta, and gamma power, all parcels significantly favored the GAM-ComBat method (p_FDR_ < 0.05). For alpha power, 46 of 68 parcels significantly favored GAM-ComBat.

**Table 3.** Table of metrics showing consistency of the 20–30 age group mean spectral power maps across the 7 studies with adequate samples within this age group, for the raw unharmonized data, and for GAM-ComBat.

	Delta		Theta		Alpha		Beta		Gamma	
	Raw	GAM-ComBat	Raw	GAM-ComBat	Raw	GAM-ComBat	Raw	GAM-ComBat	Raw	GAM-ComBat
Median Pearson R	0.94	0.98	0.88	0.96	0.98	0.99	0.80	0.94	0.86	0.99
Median Concordance Correlation Coefficient	0.84	0.88	0.67	0.81	0.89	0.90	0.59	0.82	0.70	0.96
ICC(3,k)	0.98	0.99	0.96	0.99	0.99	0.99	0.96	0.98	0.97	0.998
Median Parcel Coefficient of Variance	0.060	0.036	0.035	0.031	0.076	0.065	0.045	0.021	0.140	0.036

### 3.4 Illustration of harmonization efficacy on brain maps

**Fig. 10.**
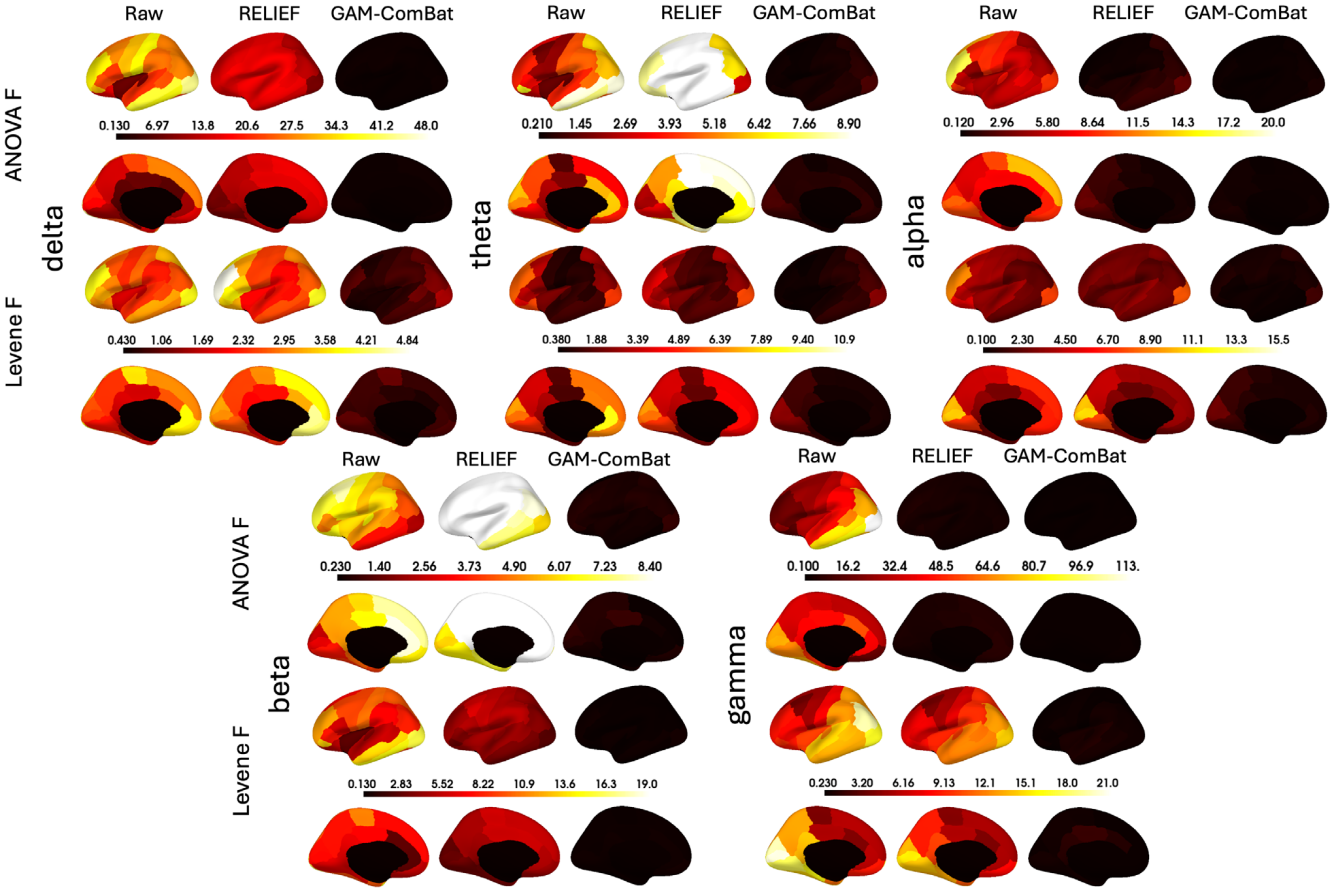
Summary plots of the ANOVA and Levene’s F tests (presented in Figs. 2 and 3) plotted on the cortical surface for one of the poorly performing methods (RELIEF) and one of the best performing methods (GAM-ComBat).

**Fig. 11.**
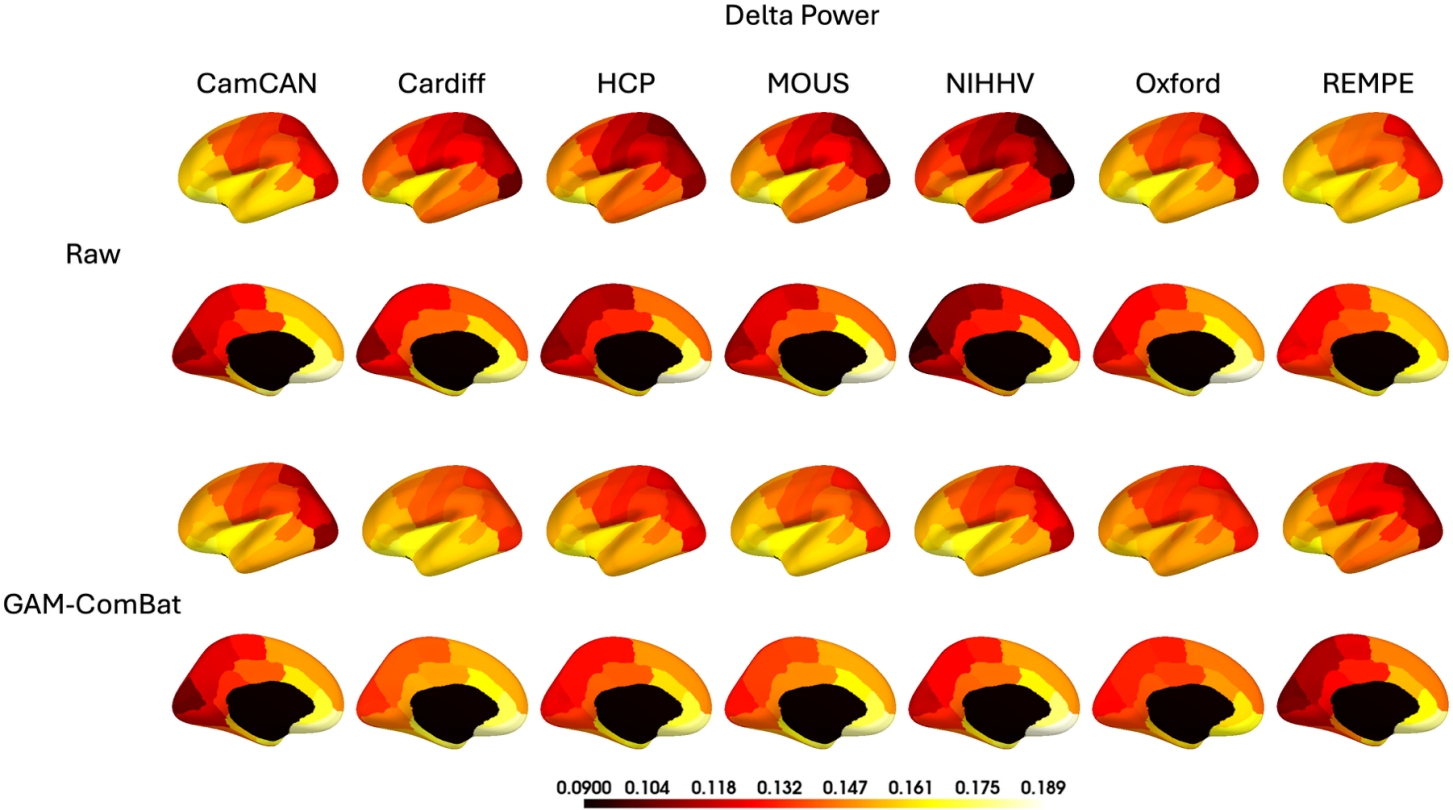
For the 20–30-year-old age group, mean delta power plotted on the cortical surface for each of the 7 sites in which this age group is well represented (N ≥ 30). Greater homogeneity in the harmonized data is evident.

Based upon the findings in the previous section, we present additional visualizations and metrics to further evaluate the efficacy of GAM-ComBat to harmonize brain maps of spectral power. In Figures 2 and 3, we plotted the ANOVA F test for equality of means and Levene’s test for equality of variances for all parcels, for all frequency bands, and for all harmonization methods. In Figure 10, we now plot those F values on the brain, for the raw data and for both GAM-ComBat (our best performing method) alongside RELIEF (a method that performed poorly in our data) to illustrate that there are in fact spatial patterns in the magnitude of study effects and that they are effectively removed by GAM-ComBat harmonization. In Figure 11, for the 20–30-year-old age group, we plot delta power across the brain for both raw and GAM-ComBat harmonized datasets, for the 7 datasets in which that age group is well represented. Similar figures for the other power bands are shown in Supplementary Figures S2–S5. Finally, in Table 3, we give median Pearson’s R and CCC between sites across all parcels, the ICC(3,k) across sites, and the median parcel CV across sites for all frequency bands.

## 4 Discussion

This manuscript, for the first time, demonstrates successful data harmonization in multi-site and multi-platform MEG. We report on four different harmonization methods, traditional ComBat, GAM-ComBat, GAM-CovBat, and RELIEF. When examining the reduction of study effects, GAM-ComBat generally performed best at removing differences in variance between sites. When examining additive or mean differences between sites, GAM-CovBat (with 100% of variance retained in the PCA) performed best, although GAM-ComBat and all CovBat versions performed similarly. Interestingly, when the site differences were plotted on brain maps, there do appear to be spatial patterns to the site differences, although the corrections provide fairly uniform control of remaining study differences across sites. While ComBat and RELIEF showed the best performance at retaining subject-level differences related to the categorical variable of task, both methods failed to retain non-linear dependencies on age. When modeling the relationship between spectral power and age, GAM-ComBat consistently produced the highest R^2^ fit values, likely due to the superior harmonization of variance across studies. Thus, we conclude that GAM-ComBat is the overall best performing method for harmonizing source-localized spectral power MEG data. Our subsequent brain maps of pre- and post-harmonized data for the 20–30 age group show the improvement in homogeneity of spectral characteristics after harmonization. While there are still differences in brain maps across sites post-harmonization, this is expected. First, sites differ on collection condition (eyes-open vs. eyes-closed rest) and also differ in their distributions of the sex of participants, both of which may affect spatial maps. Additionally, the overall distribution of ages in the 20–30-year age bin may differ across studies. The groups presented also vary in size, varying from 30 to 153 participants, which will affect variance. Finally, no harmonization method can remove all site-related signals. Nevertheless, the improvement in homogeneity and concordance metrics is striking.

It is difficult to compare our results with existing studies due to unique aspects of the data and lack of prior harmonization work with MEG data. Certainly, the superior nature of GAM-ComBat for harmonization of data with complex, non-linear dependence on age is consistent with the original description of the method, which harmonized anatomical volumes derived from MR images from 18 studies (Pomponio et al., 2020). Other studies using DTI data also demonstrated the superiority of GAM-ComBat over other harmonization methods, including CovBat (without the GAM extension) (Sun et al., 2022; Zhu et al., 2025). The failure of our modified GAM-CovBat to outperform GAM-ComBat was unexpected. However, in extensive simulations of differing types of site effects and confounding variables, the authors observed that in some situations where there are a large number of features (≥48) and sample sizes are small (≤25), CovBat may perform worse than ComBat. In addition, CovBat requires that the number of principle components (PCs) must be chosen, based on a percentage variance explained. The authors recommend 90–95%, and demonstrate in simulations that the ideal number is based in part on the number of samples per site, although they assumed equal number of samples per site. In contrast, we generally found the best performance when all PCs were retained. Given that in real-life multi-site data some site sample sizes may be small, we are reluctant to recommend this method. In the report describing the RELIEF method (Zhang et al., 2023), it substantially out-performed both ComBat and CovBat (although both methods were tested without the GAM extension). In contrast, in our data, RELIEF performed worse than all methods at removing mean and variance effects. The original report used diffusion tensor imaging (DTI)-derived metrics, which are quite different in nature from our spectral MEG data, perhaps explaining the discrepancy.

The investigation by Jaramillo-Jimenez et al. (2024), which used resting-state EEG spectral features, is the published study most similar to ours, although there are significant differences. While raw EEG and MEG data are quite similar, our MEG data are source localized, which removes environmental effects and increases the independence of individual features. While we harmonized each spectral characteristic separately, with the brain parcels used as features, Jaramillo-Jimenez et al. harmonized all spectral characteristics simultaneously, such that both EEG metrics and EEG channels comprised the features. We chose to harmonize each spectral feature separately, theorizing that sites may have environmental noise characteristics that affect various segments of the spectrum differently. Jaramillo-Jimeniz et al. observed similar performance between ComBat and GAM-ComBat in reducing site effects, in contrast to our results. They also tested HarmonizR, a variant of ComBat which compensates for missing data, and OPNComBat-GMM, a nested ComBat method, which allows for multiple batch variables and uses Gaussian mixture modeling to identify additional unknown batch effects in the data. They found that both methods outperformed both ComBat and GAM-ComBat in removal of site effects. While removal of additional batch effects (for instance, removing study, site, and platform effects) or unknown batch effects (such as those due to experimental procedures) could have utility in our investigation, the method was designed largely to compensate for bimodal feature distributions, which are not an issue with our data.

The current study has several limitations. For one, we do not test all possible harmonization methods. However, based on our findings, and the observation that the overall variance of our harmonized data is on par with the variance of individual datasets, we believe that our control of study effects is sufficient. Our recommended method, GAM-ComBat, utilized GLMGam from the python statsmodels package. The estimation of smooth terms in the GAM model is computationally intensive, and the use of a high-performance computing platform is recommended, especially for large sample sizes and large number of features. Finally, in this work, we only examined relative spectral power, rather than additional spectral metrics (such as aperiodic characteristics, frequency/bandwidth of spectral peaks, or power after removal of the aperiodic signal). While we hypothesize that peak alpha frequency may be less affected by site effects, this metric poses additional issues for harmonization. Using the FOOOF/Specparam algorithm, not all parcels for all participants will have an identified alpha peak. Participants may differ substantially in which parcels have identified alpha peaks and which do not. Since most harmonization algorithms do not support missing data of this nature, a different approach to harmonization would be required. Nevertheless, given that we only explored harmonization of spectral power, the generalizability of the results to other metrics cannot be guaranteed. We would hypothesize, however, that most source localized spectral-based metrics would respond similarly to the same harmonization method. Time-domain metrics, including amplitudes or amplitude ratios of evoked fields, or connectivity measures, may require a different strategy.

This work is the first to demonstrate that data harmonization is both possible and vital to multi-site MEG studies. Despite its numerical complexity, we recommend GAM-ComBat, due to its superior performance at reducing variance and mean differences across groups, while preserving both linear and non-linear effects of interest. GAM-ComBat is freely available under the Python implementation neuroHarmonize (Pomponio et al., 2020), and can be implemented for a wide array of data types. Given the increase in initiatives to pursue multi-site studies as a way to increase the reproducibility of neuroimaging studies, we hope that this study will be important in enabling multi-site MEG investigations.

## Supplementary Material

Supplementary Material

## Data Availability

Initial data processing was performed using the ENIGMA MEG Pipeline, available at https://github.com/nih-megcore/enigma_MEG. Code used to carry out the comparison of harmonization methods is available here: https://github.com/nugenta/MEG-Harmonize. Implementation of GAM-ComBat alone for data processed using the ENIGMA MEG Pipeline is available in the enigma_MEG github repository as enigma_harmonize.py. All data presented herein are open access datasets, either obtained by download from publicly available repositories or by request from the coordinating sites.
