# Supplementary material for: Multi-site harmonization for magnetoencephalography spectral power data
Source: Imaging Neurosci (Camb). 2026 Jan 20;4:IMAG.a.1099. doi: 10.1162/IMAG.a.1099 (PMC12820802; doi:10.1162/IMAG.a.1099)

Supplementary Table 1: Levene's test of equality of variances and ANOVA test of equality of means over the study variable, for the raw data and for each harmonization method. The mean F-value and mean p-value over all parcels is given. In each column, the lowest F-value is highlighted in dark grey.

	Levene's test of equality of variances, mean F (p) over all parcels				
	delta	theta	alpha	beta	gamma
raw	2.42 (0.04)	3.32 (0.14)	4.82 (0.001)	5.41 (<.001)	11.3 (<.001)
combat	3.05 (0.01)	1.56 (0.11)	0.67 (0.79)	2.38 (0.013)	1.10 (0.44)
relief	2.66 (0.02)	2.84 (0.05)	3.39 (0.005)	4.09 (<.001)	9.41 (<.001)
gamcombat	0.78 (0.69)	0.96 (0.60)	0.63 (0.80)	0.45 (0.93)	0.58 (0.83)
covbat 90%	0.85 (0.62)	1.78 (0.23)	1.38 (0.31)	0.67 (0.77)	0.87 (0.61)
covbat 95%	0.76 (0.71)	1.72 (0.28)	1.39 (0.30)	0.62 (0.81)	0.84 (0.65)
covbat 100%	0.65 (0.80)	1.63 (0.33)	1.40 (0.30)	0.51 (0.87)	0.82 (0.68)

	ANOVA test of equality of means, mean F (p) over all parcels				
	delta	theta	alpha	beta	gamma
raw	22.00 (<.001)	4.2 (0.03)	8.01 (<.001)	8.48 (<.001)	42.0 (<.001)
combat	9.80 (<.001)	7.10 (0.01)	0.85 (0.68)	8.31 (<.001)	2.50 (0.041)
relief	13.49 (<.001)	7.99 (0.005)	1.22 (0.47)	8.88 (<.001)	2.76 (0.02)
gamcombat	0.48 (0.91)	0.53 (0.88)	0.33 (0.98)	0.28 (0.99)	0.38 (0.97)
covbat 90%	1.58 (0.30)	1.01 (0.54)	0.87 (0.67)	0.67 (0.76)	1.03 (0.52)
covbat 95%	0.57 (0.83)	0.74 (0.71)	0.48 (0.89)	0.57 (0.83)	0.58 (0.83)
covbat 100%	0.38 (0.95)	0.41 (0.91)	0.22 (0.99)	0.19 (1.00)	0.30 (0.99)

Supplementary Table 2: Median partial R2 values, showing the additive effect of site present in the raw data and after each harmonization method is performed. The lower pane gives the mean log variance ratio, showing the multiplicative site effect present in the raw data and after each harmonization method is performed. In each column, the lowest value (indicating the lowest residual study effects) is highlighted in dark grey.

	Median partial R2 for site				
	delta	theta	alpha	beta	gamma
raw	0.0650	0.0187	0.0477	0.0325	0.1990
combat	0.1285	0.1005	0.0064	0.1021	0.0335
relief	0.1620	0.1080	0.0088	0.1130	0.0357
gamcombat	0.0014	0.0023	0.0022	0.0011	0.0022
covbat 90%	0.0049	0.0041	0.0048	0.0027	0.0060
covbat 95%	0.0017	0.0025	0.0032	0.0230	0.0035
covbat 100%	0.0011	0.0013	0.0014	0.0007	0.0020
	Mean absolute |logVR| across sites				
	delta	theta	alpha	beta	gamma
raw	0.2194	0.2445	0.2542	0.2677	0.3332
combat	0.2506	0.1992	0.0854	0.1923	0.1181
relief	0.1985	0.2100	0.2331	0.2767	0.3084
gamcombat	0.0856	0.0848	0.0817	0.0581	0.0758
covbat 90%	0.0954	0.1205	0.1245	0.0725	0.0947
covbat 95%	0.0889	0.1207	0.1470	0.0678	0.0919
covbat 100%	0.0796	0.1120	0.1209	0.0589	0.0909

Supplementary Figure 1: For a single representative parcel (right transverse temporal cortex) we show the distribution of data from each study after removing the effects of sex, task (eyes open/eyes closed), age, age^2, and age^3. Raw unharmonized data is shown (top left) along with all tested harmonization methods. Differences in the site means and variances can be appreciated in the raw data and ComBat and RELIEF methods, with greater uniformity in the GAM-ComBat and CovBat methods.

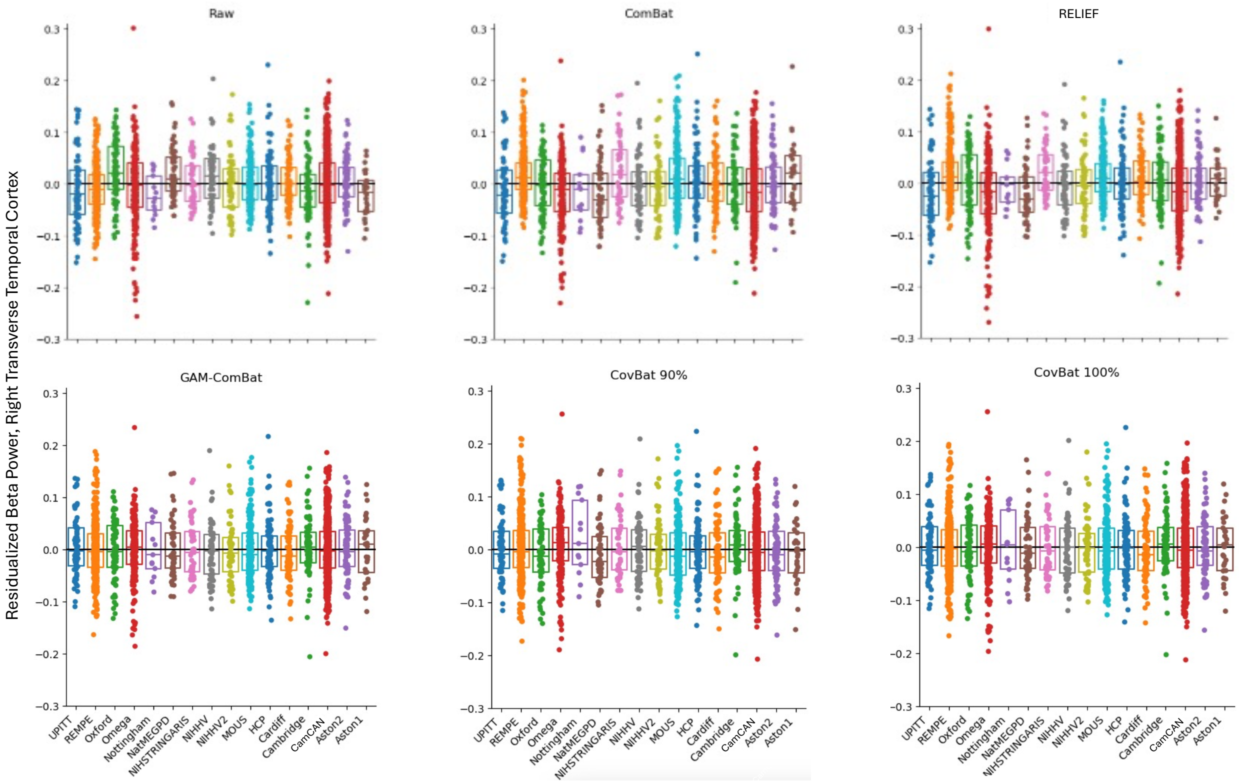

Supplementary Figure 2: For the 20–30 year old age group, mean theta power plotted on the cortical surface for each of the 7 sites in which this age group is well represented (N>=30).

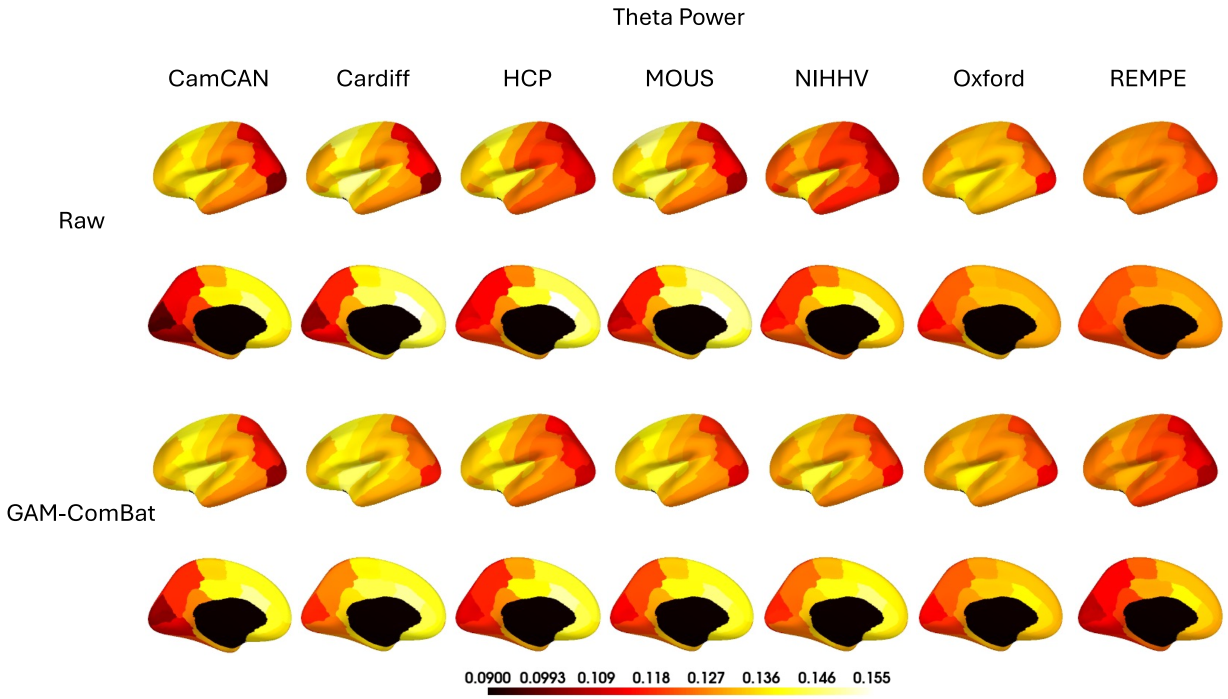

Supplementary Figure 3: For the 20–30 year old age group, mean alpha power plotted on the cortical surface for each of the 7 sites in which this age group is well represented (N>= 30).

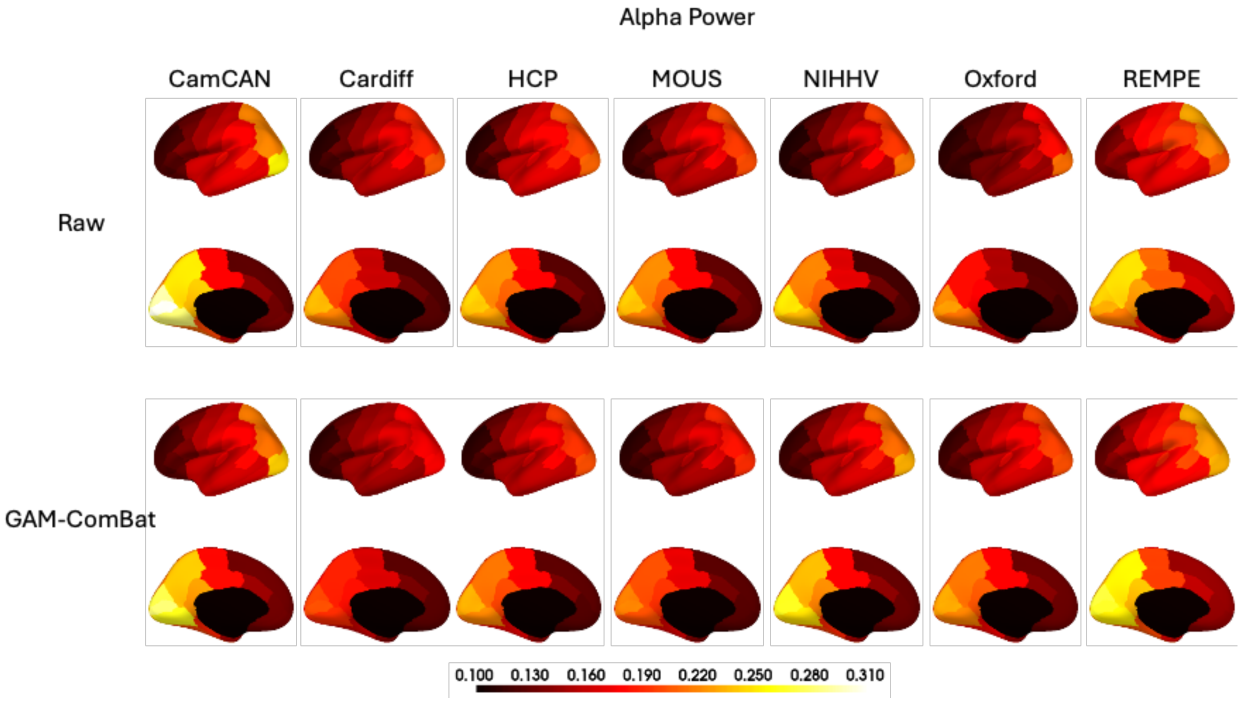

Supplementary Table 4: For the 20–30 year old age group, mean beta power plotted on the cortical surface for each of the 7 sites in which this age group is well represented (N>= 30).

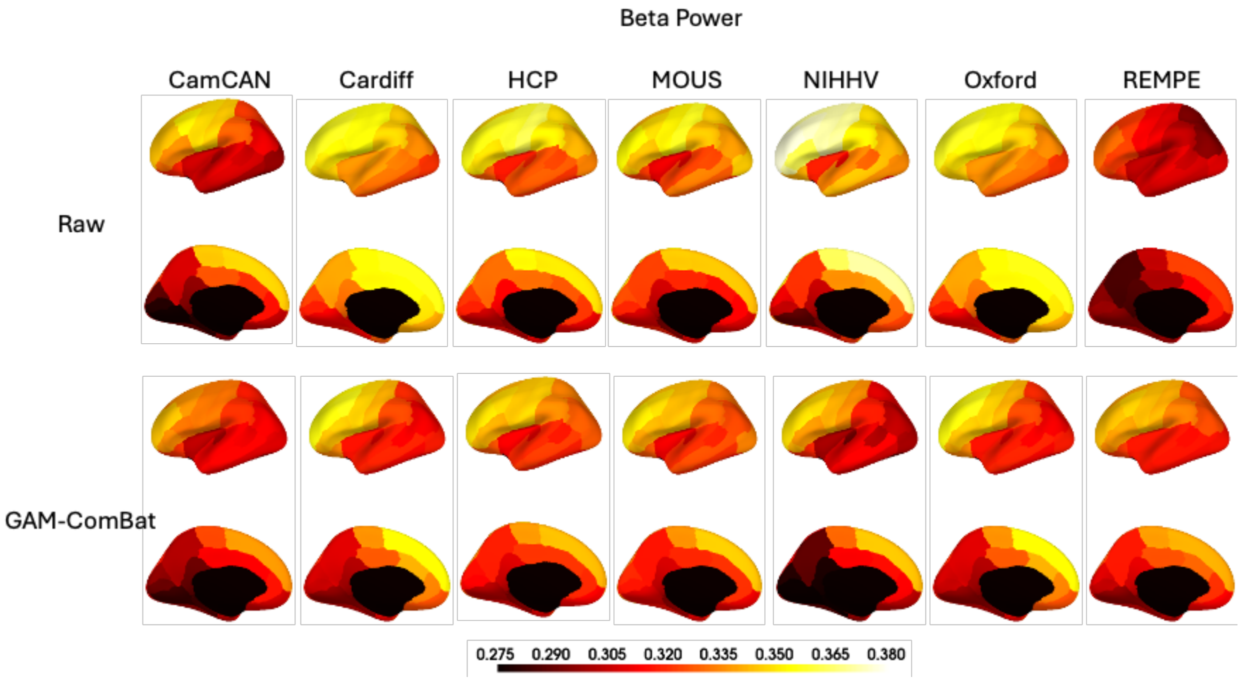

Supplementary Figure 5: For the 20–30 year old age group, mean gamma power plotted on the cortical surface for each of the 7 sites in which this age group is well represented (N>= 30).

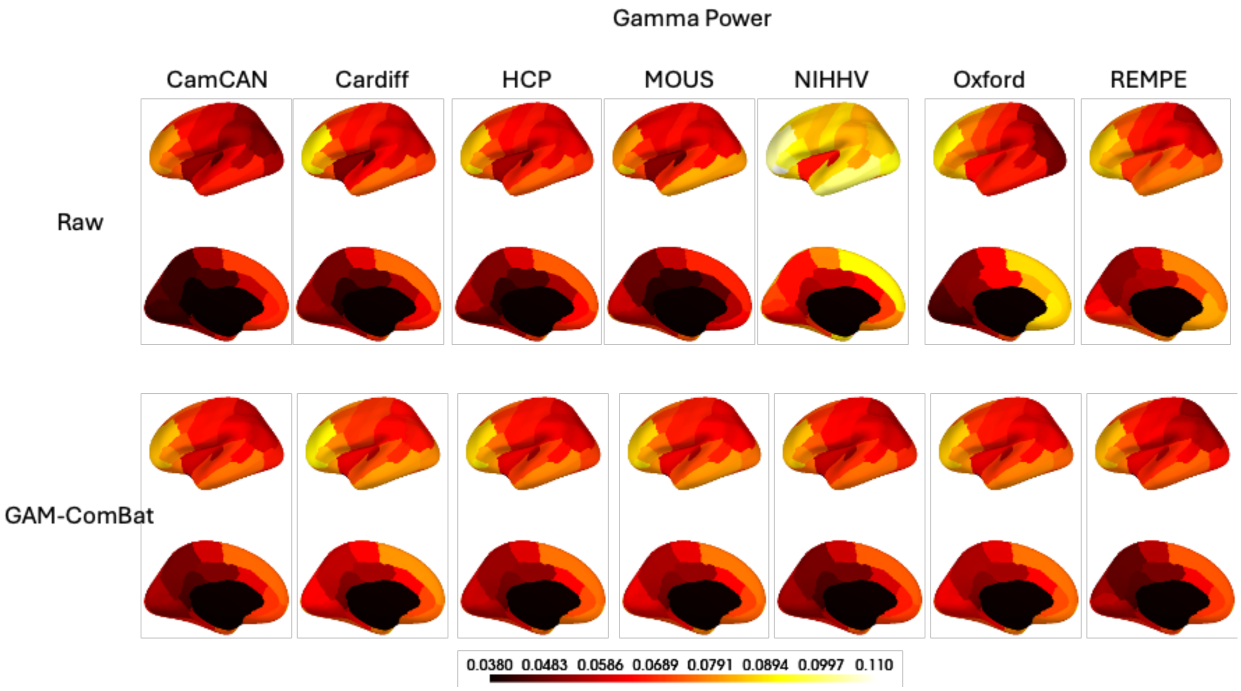

Supplement: Supplementary Material [file IMAG.a.1099_supp.pdf]
